# Synovial Periprosthetic Infection Markers Show High Variability in Different Clinical and Microbiological Settings

**DOI:** 10.3390/jcm15010052

**Published:** 2025-12-21

**Authors:** Joachim Ortmayr, Jennifer Straub, Klemens Vertesich, Irene Katharina Sigmund, Christoph Böhler, Reinhard Windhager, Kevin Staats

**Affiliations:** Department of Orthopaedics and Trauma Surgery, Division of Orthopaedics, Medical University of Vienna, Währinger Gürtel 18-20, 1090 Vienna, Austriakevin.staats@meduniwien.ac.at (K.S.)

**Keywords:** periprosthetic joint infection (PJI), synovial fluid, white blood cell count (WBC), polymorphonuclear cells, PMN

## Abstract

**Background/Objectives:** Accurately diagnosing a periprosthetic joint infection (PJI) represents a challenging and complex task. Especially in the case of low-grade infections, important diagnostic modalities may be inconclusive and synovial markers such as white blood cell count (WBC) and polymorphonuclear percentage (PMN) gain relevance. We therefore aim to assess WBC and PMN in different clinical and microbiological settings. **Methods:** We performed a retrospective analysis of 115 patients with a diagnosed PJI. WBC and PMN were compared between patients with low- and high-virulent infections, negative and positive histology, symptom duration ≤ 4 weeks and >4 weeks, and positive and negative pathogen detection. **Results:** Synovial WBC was significantly higher in patients with successful pathogen detection (42.44 [87.0] G/L vs. 16.35 [32.0] G/L; *p* < 0.01), as was PMN (86.0 [60.0]% vs. 91.0 [89.0]%; *p* < 0.01). PJIs with high-virulent pathogens showed higher WBC compared to low-virulent pathogens (58.27 [102.0] G/L vs. 27.27 [46.0] G/L; *p* < 0.01). Patients with onset of symptoms ≤ 4 weeks demonstrated higher WBC (58.27 [112.0] G/L vs. 16.42 [46.0] G/L]; *p* < 0.01) as well as higher PMN (91.5 [9.0]% vs. 88.0 [20.0]%); *p* = 0.042). Cases with negative histology showed significantly lower WBC (16.73 [44.0] G/L vs. 42.86 [87.0] G/L; *p* < 0.01) and lower PMN (86.0 [67.0]% vs. 91.0 [9.0]%; *p* = 0.036). **Conclusions:** WBC and PMN show high variability and appear to be influenced by virulence, histology, onset of symptoms, and pathogen detection.

## 1. Introduction

With higher life expectancy and, consequently, an increasing demand for total hip and knee arthroplasty, the prevalence of major complications such as periprosthetic joint infection (PJI) is expected to rise [1,2,3]. The incidence of PJI is 1.55% within the first 2 years and decreases to 0.46% between 2 and up to 10 years [4]. Treatment involves extended revision surgery, which is associated with higher mortality rates, prolonged hospital stays, and high failure rates [5,6,7,8,9,10].

Synovial markers white blood cell count (WBC) and polymorphonuclear percentage (PMN), both included in the established diagnostic guidelines, are important tools in the standard diagnostic workup for PJI. However, diagnostic accuracy may vary, depending on external factors such as the underlying pathogen. Kheir et al. could detect lower values across all established diagnostic markers when infected with low-virulent organisms such as coagulase-negative Staphylococcus or Cutibacterium acnes [11]. Respective cases may show minor elevations of synovial WBC and PMN and thus potentially be misdiagnosed as they do not exceed pre-determined cut-off values. In addition, patients often exhibit unspecific clinical symptoms such as joint pain without the typical signs of inflammation and other diagnostic tools such as culture demonstrate limited diagnostic reliability with reported false-negative rates up to 50% in case of low-virulent pathogens [12,13,14,15,16]. The distinction between PJI and aseptic failure thus remains challenging.

Given these limitations within the current diagnostic framework, we aim to assess WBC and PMN in different diagnostic settings, considering histology, onset of symptoms, pathogen detection and virulence. We further assess if there are differences in WBC and PMN in patients with pathogen detection in different detection methods, used preoperatively and intraoperatively.

## 2. Materials and Methods

**Figure 1:**
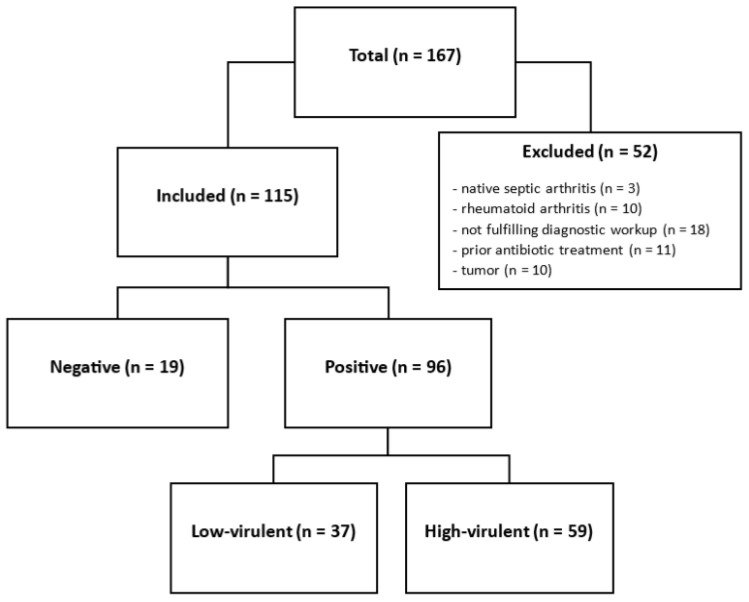
Flowchart of the included patient cohort.

After Institutional ethics committee approval (Nr. 1259/2021), we reviewed our institutional database of revision total joint arthroplasty for patients who underwent revision hip or knee arthroplasty for PJI at our center between March 2016 and March 2023. PJI was defined by the EBJIS criteria [17], using the thresholds of the infection-likely group. Cases with either WBC or PMN values surpassing the threshold of 1500 cells/µL or 65% were included. Data obtained from patients included demographic data, clinical appearance, synovial WBC and PMN, and microbiological and histopathological analysis. Patients treated with antibiotics two weeks prior to diagnostic evaluation, native joint arthritis, not fulfilling the whole diagnostic workup according to the EBJIS guidelines, underlying inflammatory arthritis (e.g., rheumatoid arthritis), and arthroplasty due to tumor resection were excluded. A total of 115 patients treated with a revision surgery of either the hip or knee joint were identified. A total of 10 patients were excluded due to rheumatoid arthritis, 11 patients due to prior antibiotic treatment, and 10 patients due to arthroplasty due to tumor resection (Figure 1). Further additional demographic details for the participants can be found in Table 1.

Preoperative joint aspiration was performed under aseptic conditions and aspirates were sent for microbiological culture, either broad-spectrum Polymerase Chain Reaction (PCR) (SepsiTest™-UMD Kit IVD (Molzym, Bremen, Germany)) or multiplex PCR (BIOFIRE^®^ Joint Infection Panel, FilmArray^®^ Torch System (bioMérieux, Salt Lake City, UT, USA)) as well as synovial fluid analysis for WBC and PMN. Synovial WBC values are reported in G/L (1 G/L = 1000 cells/µL). Intraoperatively, five periprosthetic tissue specimens for culture and histological assessment were harvested at locations with macroscopically highest inflammatory changes; prosthetic components were packed in airtight containers filled with 0.9% saline for sonication. Cultures were held for at least 14 days. Pathogens were divided according to their virulence in high- or low-virulent pathogens [18]. Histology was assessed via the synovial-like interface membrane (SLIM)-classification. SLIM I (particle-induced) and SLIM IV (indifferent) were classified as negative histology, whereas SLIM II (infectious) and SLIM III (combination of particle-induced and infectious) were rated as positive histology. Clinical appearance was assessed as onset of symptoms (≤4 weeks vs. >4 weeks). 

WBC and PMN were compared between patients with low- and high-virulent infections, negative and positive histology, symptom duration ≤ 4 weeks and >4 weeks, and positive and negative pathogen detection. We further assessed whether there were differences when pathogen detection occurred in a certain detection method. We further categorized patients into two groups based on their WBC (>3.0 G/L vs. ≤3.0 G/L) and assessed whether there is a significant association between exceeding this limit and the above-mentioned clinical and diagnostic characteristics.

### Statistical Analyses

Statistical analyses were performed using SPSS version 27.0 (IBM, Armonk, New York, NY, USA). Descriptive statistics were presented as median (interquartile range). Normality of the data was assessed using the Shapiro–Wilk test for smaller samples and the Kolmogorov–Smirnov test for larger samples. WBC and PMN were compared for statistical significance using the Wilcoxon rank sum test for non-parametric data and the unpaired *t*-test for parametric data. To compare WBC and PMN between different detection methods, we used the Kruskal–Wallis rank sum test, and for post hoc pairwise comparisons, we employed the Bonferroni-adjusted Wilcoxon rank sum test. To determine whether there was a significant association between WBC > 3.0 and ≤3.0 G/L and clinical and diagnostic characteristics, we used the chi-squared test. To better quantify the strength of the association, we calculated the Odds Ratio (=OR). When one or more cells of the table have a count of zero, the Haldane–Anscombe correction was used. An alpha level of 0.05 was used to determine significance.

## 3. Results

### 3.1. Pathogen Detection

**Figure 2:**
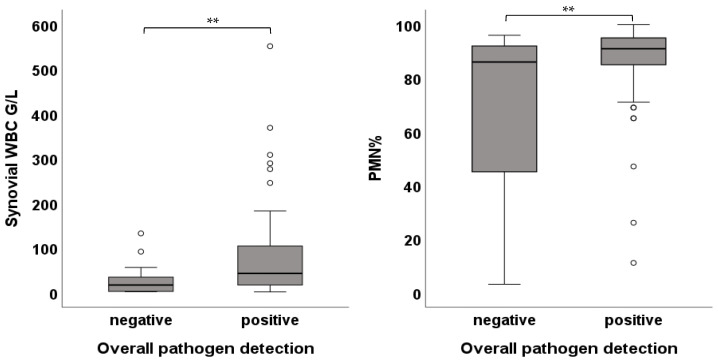
Box plots showing the cell count and PMN values in cases with negative and positive pathogen detection (** *p* < 0.01).

A pathogen was detected in 83.5% (n = 96) of all cases. Synovial WBC was significantly higher in patients with successful pathogen detection (42.44 [87.0] G/L vs. 16.35 [32.0] G/L; *p*-value < 0.01), as was PMN (86.0 [60.0]% vs. 91.0 [10.0]%; *p*-value < 0.01) (Figure 2).

**Figure 3:**
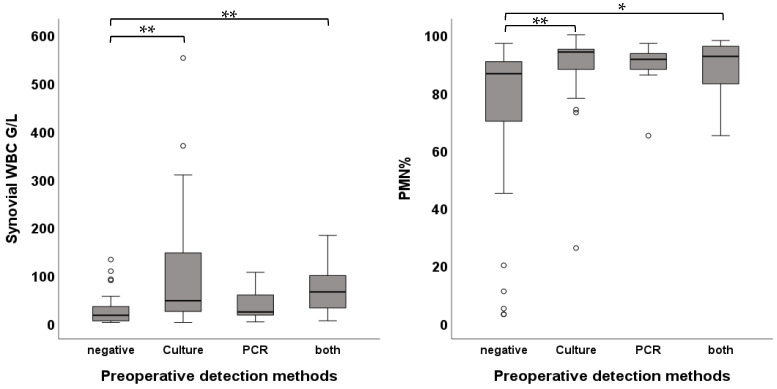
Box plots showing the comparison of WBC and PMN between preoperative detection methods (* *p* < 0.05, ** *p* < 0.01).

Preoperative pathogen detection in synovial fluid, either by culture or PCR, was successful in 67.0% (n = 77). In 37.4% (n = 43) of the cases, pathogens were detected only in culture, in 7.8% (n = 9) only in PCR, and in 21.7% (n = 25) in both. Patients with a pathogen detected only in PCR showed no significant difference in synovial WBC compared to pathogen-negative cases (22.97 [46.0] G/L vs. 16.35 [32.0] G/L); *p*-value = 1.0), while cases with pathogen isolation only in culture (46.41 [126.0] G/L; *p*-value < 0.01) and positive in both culture and PCR (64.71 [70.0] G/L; *p*-value < 0.01) showed significantly higher synovial WBC when compared to pathogen-negative cases (16.35 [32.0] G/L). Comparisons between PCR-positive (22.97 [46.0] G/L]) and culture-positive (46.41 [126.0] G/L; *p*-value = 0.7) or positive in both (64.71 [70.0] G/L; *p*-value = 0.943) showed no significant differences. When comparing PMN between these groups, culture-positive cases (94.0 [9.0]%; *p*-value = 0.01) and cases positive in both PCR and culture (92.50 [13.0]%; *p*-value = 0.024) had significantly higher PMN when compared to pathogen-negative cases (86.50 [21.0]%). No significant difference between pathogen-negative cases (86.50 [21.0]%) and PCR-positive cases (91.50 [7.0]%; *p*-value = 0.618) was noted (Figure 3).

**Figure 4:**
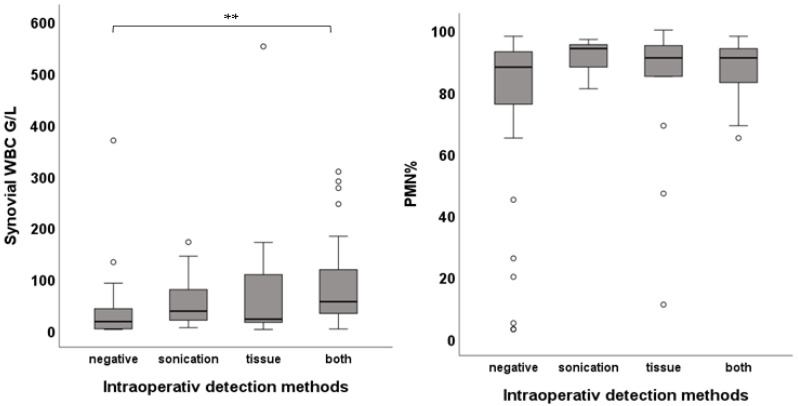
Box plots showing the comparison of WBC and PMN between intraoperative detection methods (** *p* < 0.01).

Successful intraoperative pathogen detection was given in 68.7% (n = 79). Cases only positive in tissue culture accounted for 13.9% (n = 16), cases only positive in sonication for 17.4% (n = 20) and those positive in both for 37.4% (n = 43). When comparing synovial WBC between cases only positive in tissue culture (21.33 [95.0] G/L; *p*-value = 0.723) and cases only positive in sonication (36.88 [66.0] G/L; *p*-value = 0.29) to pathogen-negative cases (16.35 [32.0] G/L]), there were no significant differences. Cases positive in both showed significantly higher WBC compared to pathogen-negative ones (55.32 [90.0] G/L vs. 16.35 [32.0] G/L; *p*-value < 0.01). No differences were found in PMN (Figure 4).

### 3.2. Clinical Appearance, Histology, Virulence

**Figure 5:**
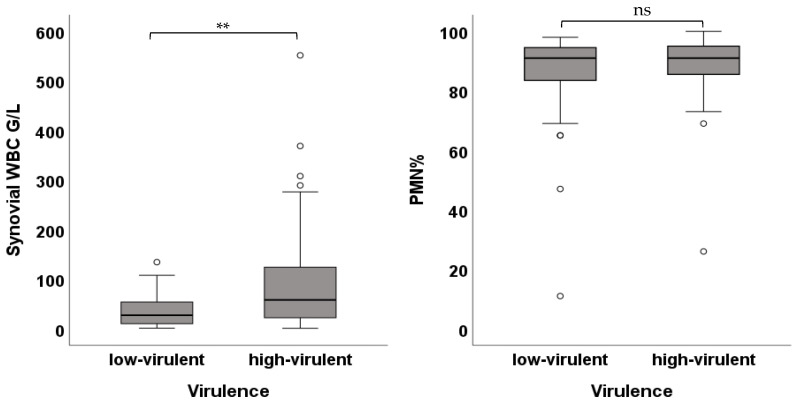
Box plots showing the WBC and PMN of infections with high-virulence compared to low-virulence pathogens (** *p* < 0.01, ns = not significant).

**Figure 6:**
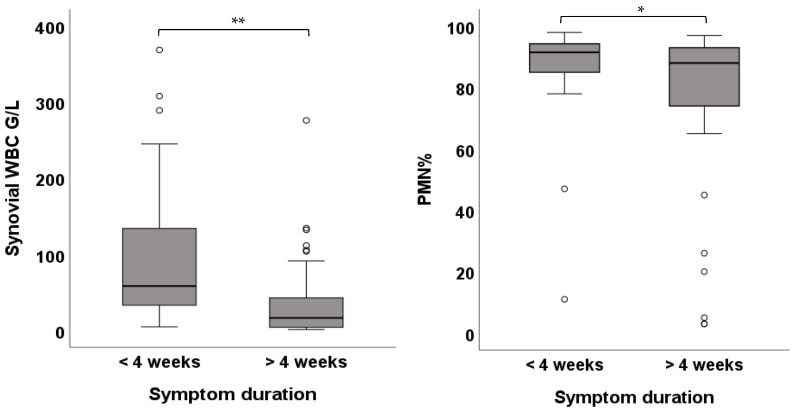
Box plots showing the WBC and PMN of cases with onset of symptoms ≤ 4 weeks and >4 weeks (* *p* < 0.05, ** *p* < 0.01).

**Figure 7:**
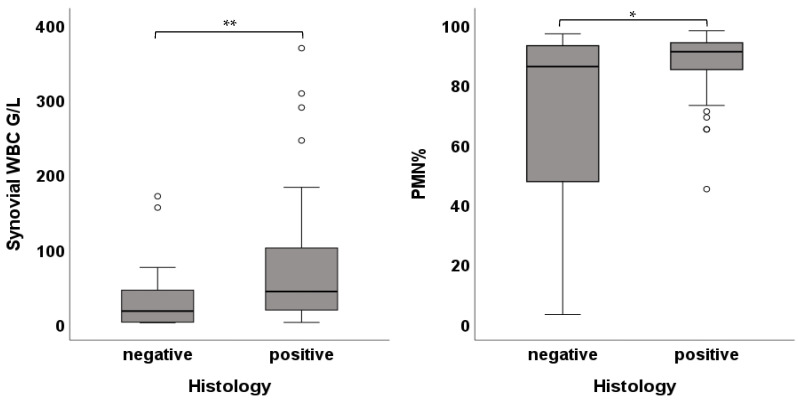
Box plots showing the WBC and PMN of infections with positive and negative histology (* *p* < 0.05, ** *p* < 0.01).

PJIs with high-virulent pathogens showed higher WBC compared to low-virulent pathogens (58.27 [102.0] G/L vs. 27.27 [46.0] G/L; *p*-value < 0.01). There was no difference between low- and high-virulent infections in PMN (91.0 [13.0]% vs. 91.0 [10.0]%; *p*-value = 0.631) (Figure 5). Patients with an onset of symptoms ≤ 4 weeks demonstrated higher WBC (58.27 [112.0] G/L vs. 16.42 [46.0] G/L; *p*-value < 0.01) as well as higher PMN (91.50 [9.0]% vs. 88.0 [20.0]%; *p*-value = 0.042) (Figure 6). Cases with negative histology showed significantly lower WBC (16.73 [44.0] G/L vs. 42.86 [87.0] G/L; *p*-value < 0.01) and lower PMN (86.0 [67.0]% vs. 91.0 [9.0]%; *p*-value = 0.036) (Figure 7). Comparison among different groups is shown in Table 2.

### 3.3. Associations Between WBC and Clinical and Diagnostic Characteristics

We could show statistically significant associations between exceeding the WBC cut-off value of 3.0 G/L and histology (*p*-value < 0.01), symptom duration (*p*-value < 0.01), and overall pathogen detection (*p*-value < 0.01). The odds for WBC not exceeding 3.0 G/L were 1.21 times higher given an infection with a low-virulent pathogen, 9.37 times higher in the presence of negative histology, 31.67 higher given a symptom duration > 4 weeks, and 5.87 times higher in cases without successful pathogen detection. Preoperatively, cases with culture-positive results (OR = 2.6), PCR-positive (OR = 2.13), and positive in both (OR = 14.21) showed higher odds for surpassing the threshold of 3.0 G/L than pathogen-negative cases. Intraoperatively, the odds for sonication-positive cases were 16.25 times higher, 5.77 times higher for tissue-positive cases, and 7.88 times higher for both positive cases to surpass 3.0. G/L than for pathogen-negative cases.

## 4. Discussion

Accurately diagnosing periprosthetic joint infection (PJI) is critical for further treatment strategies and ultimately functional outcome after surgery [19]. However, this is often difficult, especially in so-called low-grade infections. Thus, surgeons must rely on a combination of diagnostic markers including synovial WBC and PMN for clinical decision-making [20]. Both are widely used, cost-efficient diagnostic tools, integrated into current International Consensus Meeting and European Joint Infection Society criteria [17,21]. Thresholds for WBC and PMN are set at 3.0 G/L and 80% to confirm PJI. However, patients may exhibit reduced elevation of synovial markers, potentially creating a gray area for the distinction between aseptic and septic complication when using traditional cut-off values [22]. To allow better interpretation in different diagnostic settings, we assessed differences in the synovial markers, WBC and PMN, considering histology, onset of symptoms, and virulence. We further assess whether there are differences in WBC and PMN if pathogen detection occurred in a certain method preoperatively and intraoperatively.

The main finding of the study is the high variability in WBC and PMN values, with both being strongly dependent on the type of pathogen, detection method, histology, and clinical appearance. Clinicians should be aware that low-virulent infections, delayed symptom onset, or negative histology can lead to lower marker values. These markers should therefore be interpreted within the broader diagnostic context rather than relying on strict cut-off values alone.

Regarding pathogen virulence, several studies indicate that alteration of synovial markers is highly dependent on virulence and the underlying pathogen. Kheir et al. could detect lower values across all established diagnostic markers when infected with low-virulent organisms such as coagulase-negative Staphylococcus or Cutibacterium acnes [11]. Deirmengian et al. observed significantly lower median synovial fluid CRP levels for less virulent organisms [23]. Our data corroborate these findings as low-virulent infections showed reduced WBC. Pathogens in chronic periprosthetic infections typically form a biofilm around the implant that provides protection from surrounding inflammatory cells, resulting in a reduced immune response [24,25]. Correspondingly, in the chronic setting, patients may suffer from a subclinical course with reduced pain. Accordingly, we could observe reduced WBC and PMN values in patients with onset of symptoms > 4 weeks. Furthermore, as indicated by reduced inflammatory activity, cases with negative histology demonstrate lower WBC and PMN values.

Up to 50% of patients are wrongly classified as culture-negative, contributing to diagnostic uncertainty [14,26]. Within our cohort, pathogen-negative cases exhibit significantly lower WBC and PMN. This supports findings from previous reports that observed similar results in culture-negative cases [11]. Molecular techniques such as broad-spectrum and multiplex PCR have improved diagnostic accuracy and offer increased sensitivity for the detection of pathogens, particularly low-virulent and biofilm-associated organisms. It is therefore able to identify infections that may be missed by conventional culture methods [27,28]. However, it is more susceptible to contamination from human DNA and low-virulent bacteria that are part of normal skin flora and thus to false-positive results [29]. A distinction between contaminants and clinically relevant pathogens cannot be made. Currently, it is not included in any of the established guidelines. In our cohort, there was no difference in synovial WBC or PMN between pathogen-negative and PCR-positive cases. This raises the question of whether patients with a PJI only positive in PCR show low values of synovial markers or whether PCR gives a higher rate of false-positive pathogen results. The higher sensitivity is at the cost of reduced specificity, requiring careful interpretation of PCR-only positive cases to balance the risk of missed infections against the potential for findings of uncertain relevance. Cases, either culture-positive only or both PCR- and culture-positive, showed significantly higher WBC and PMN compared to pathogen-negative cases. When comparing synovial markers among intraoperatively used detection methods, WBC was significantly higher in cases with positive culture in tissue and sonication together compared to negative cases.

In line with this data, there is a statistically significant association between exceeding the threshold WBC value of 3.0 G/L and positive histology, symptom duration under 4 weeks, and positive overall pathogen detection. In this study, there were higher odds for WBC value < 3.0 G/L in cases with negative histology, low-virulent pathogens, symptom duration > 4 weeks, and no pathogen detection. Regarding detection methods, the odds of surpassing the WBC threshold of 3.0 G/L was especially high in cases where pathogen detection was accomplished preoperatively in both culture- and PCR-positive and culture-positive only, and intraoperatively in all three positive groups compared to negative cases. One potential explanation can be a higher bacterial load in these cases.

Of particular interest is that pathogen-negative cases and cases with pathogen detection in PCR showed lower values of synovial markers. Given the poor performance of traditional culture and the discrepancies in WBC and PMN shown in our study, research is focusing on finding more effective methods such as microbial-DNA-detecting techniques [30,31,32]. They may potentially reduce the incidence of culture-negative cases with a detection rate in some studies up to 81.7% [33]. As lower synovial markers can be expected in these cases, current cut-off values may have to be re-examined. However, further research is warranted as to whether these cases show relevant differences in marker elevation compared to culture-positive cases.

There are a number of limitations of this study that should be considered. First, its retrospective design and limited sample size are susceptible to inherent biases typically observed in such studies. Information about exclusion criteria such as rheumatoid arthritis or prior antibiotic treatment relies on precise documentation, and thus, some cases could be missed. Tissue sampling was variable among the surgeons and thus potentially influences quality for culturing and histological analysis. Second, aspiration prior to surgery was not standardized and, in some cases, was performed at different time points, creating a potential selection bias. Third, diagnostic criteria were adapted over the years, with newer tools integrated into the diagnostic algorithm. This, however, could lead to a higher exclusion rate of older cases as these tools were not available at that time. Additionally, as inclusion required WBC or PMN values above the EBJIS infection-likely thresholds, our cohort is inherently biased toward higher synovial marker values, which may have influenced overall marker distributions and subgroup comparisons. This approach was chosen deliberately, as values below these thresholds make infection highly unlikely within the EBJIS framework, and our intention was to illustrate the substantial variability of synovial markers in PJI cases.

## 5. Conclusions

The presented data contribute to a better understanding of WBC and PMN in different clinical and diagnostic settings. Both synovial markers exhibit considerable variability across clinical and microbiological contexts, highlighting the need to interpret these values within the broader diagnostic framework.

## Figures and Tables

**Table 1:** Patient demographics.

**Demographics**	
Age *	71.1 (sd = 12.4)
Sex	
Male	55
Female	60
Joint	
Hip	40
Knee	75

* The values are given as the mean. The values are given as the number of patients.

**Table 2:** Comparison of WBC and PMN among different groups.

Variable *			*p*-Value ^†^
Overall Pathogen Detection	negative	positive	
WBC G/L	16.35 [32.0]	42.44 [87.0]	<0.01
PMN%	86.0 [60.0]	91.0 [10.0]	<0.01
Histology	negative	positive	
WBC G/L	16.73 [44.0]	42.86 [87.0]	<0.01
PMN%	86.0 [67.0]	91.0 [9.0]	=0.036
Symptom Duration	>4 weeks	≤4 weeks	
WBC G/L	16.42 [46.0]	58.27 [112.0]	<0.01
PMN%	88.0 [20.0]	91.50 [9.0]	=0.042
Virulence	low	high	
WBC G/L	27.27 [46.0]	58.27 [102.0]	<0.01
PMN%	91.0 [13.0]	91.0 [10.0]	=0.631

* The values are given as the median (interquartile range). ^†^ Wilcoxon rank sum test for non-parametric data and the unpaired *t*-test for parametric data was used to compare WBC and PMN between the groups.

## Data Availability

The data presented in this study are available on request from the corresponding author.
